# Copper Intoxication in South American Camelids—Review of the Literature and First Report of a Case in a Vicuña (*Vicugna vicugna*)

**DOI:** 10.1007/s12011-024-04102-x

**Published:** 2024-02-29

**Authors:** H. Marahrens, K. von Dörnberg, V. Molnár, K. M. Gregor, E. Leitzen, A. von Altrock, A. Polifka, M. Ganter, M. G. Wagener

**Affiliations:** 1 Clinic for Swine and Small Ruminants, Forensic Medicine and Ambulatory Service, University of Veterinary Medicine, Foundation, Hannover, Germany; 2 Hannover Adventure Zoo, Hannover, Germany; 3 Department of Pathology, University of Veterinary Medicine, Foundation, Hannover, Germany

## Abstract

Copper (Cu), an essential trace element in the metabolism of mammals, plays a central role in various metabolic processes. However, overdosing can lead to severe symptoms and even fatalities. Chronic Cu intoxication continues to be a problem in grazing and domestic animals, with sheep being particularly sensitive. There are few comparative studies on its impact on South American camelids (SACs). Therefore, this work presents the results of literature research combined with a case report on a 3-year-old female vicuña (*Vicugna vicugna*) presented to the clinic from a zoological garden in northern Germany. The animal showed reduced food intake, recumbency, bruxism, icteric mucous membranes and sclera. Auscultation revealed atony of the third compartment and the digestive tract. Similar to cases described in the literature, the animal showed rapid deterioration of its condition with unspecific symptoms of liver failure and rapid death. However, in contrast to descriptions in sheep, clinical icterus has not been previously reported in cases of other SACs. Laboratory findings from EDTA and serum samples revealed neutrophilia with a left shift, hypoproteinaemia, lymphopaenia, azotaemia, elevated levels of creatine kinase (CK), aspartate aminotransferase (AST) and glutamate dehydrogenase (GLDH) in the serum. Hyperbilirubinaemia and significantly elevated serum and liver Cu levels were observed. Subsequent blood samples from the remaining vicuñas and alpacas in the same enclosure showed no remarkable abnormalities. To the best of the authors’ knowledge, this case report represents the first documented case of Cu intoxication specifically in vicuñas.

## Introduction

Copper (Cu) poisoning is a known problem in veterinary medicine, impacting various mammals kept in human care and has been frequently described for farm animals like small ruminants [1–5], cattle [6–8], pigs [9, 10] and horses [11–13]. Monogastric animals generally seem to be less affected by the consequences of excessive Cu intake, in contrast to ruminants [14, 15]. Among these, the high sensitivity of sheep to Cu accumulation has been particularly well studied and described [1, 14–19], while the literature on Cu poisoning in South American camelids (SACs) remains limited. This study aims to provide an overview of the existing reports on this species, focusing on clinical, laboratory and pathological findings in a vicuña, alongside laboratory findings from other SACs kept in the same enclosure, as this case was presented to the Clinic for Swine and Small Ruminants at the University of Veterinary Medicine Hannover, Foundation, Germany.

### Copper Intoxication in Sheep

It is presumed that sheep (*Ovis aries*) are unable to increase the Cu excretion rate via the bile in cases of excessive intake [1], while also breed-specific variations in Cu plasma levels [20] and liver accumulation [21] have been documented. Chronic Cu poisoning is reported more frequently as acute poisoning, and the ovine liver can store large Cu quantities without displaying symptoms during a long subclinical pre-haemolytic phase lasting weeks to months [22]. Next to primary intoxication caused by excessive ingestion, other factors such as a low intake of antagonists like molybdenum (Mo), sulphur (S), zinc (Zn) or iron (Fe) that increases Cu absorption following oral ingestion in general or the ingestion of hepatotoxic substances that negatively affect liver metabolism and thus excretion play an important role. Both pathways can lead to hepatocyte necrosis, release of Cu into the bloodstream and a haemolytic crisis due to the toxicity of Cu ions on hepatocytes and erythrocyte membranes, in addition to the formation of methaemoglobin [14]. Clinical findings following chronic administration are rather nonspecific due to the haemolytic crises resulting in haemoglobinuria, icterus and anaemia, alongside disorders in the central nervous system and renal problems due to haemoglobin flooding [14, 22]. Depression, anorexia, apathy, bruxism and excessive thirst are listed as clinical symptoms, with a high risk of mortality for affected animals [1].

### Copper Intoxication in South American Camelids

In general, SACs are classified in terms of their sensitivity to Cu as being intermediate between sheep and other ruminants [23]. In 2001, Carmalt et al. described the case of a 10-year-old female alpaca (*Vicugna pacos*) that was presented with lateral recumbency and dyspnoea [24]. Despite treatment with antibiotics, infusion and oxygen supply, the condition of the animal deteriorated, and it died the following day suffering from tonic-clonic convulsions. Urination and defaecation in this case were described to be normal. Necropsy of this animal revealed necrotic periacinal changes of hepatocytes and an elevated liver Cu content of 640.5 mg/kg dry matter (DM), while Mo was 6.16 mg/kg DM. The liver contents of Fe, magnesium (Mg), Zn and manganese (Mn) were inconspicuous. The suspected reason for the increased Cu content in the liver in this case was intake of a pelleted feed with a Cu content of 20–30 mg/kg DM which was fed during a 4-year period.

Junge and Thornburg described the cases of Cu intoxication in four llamas from a zoo enclosure that had been noticed exhibiting signs of apathy and lateral recumbency before they died rapidly in 1989 [25]. The histological necropsy also revealed severe necrotic changes of hepatocytes, partly with bile duct proliferation, while the Cu content was measured in three livers from llamas at the age of 8 years, 4 months and 8 months reporting 1700 mg/kg DM, 870 mg/kg DM and 847 mg/kg DM, respectively. Previously collected blood samples from these animals also showed severely increased activities of aspartate aminotransferase (AST), lactate dehydrogenase (LDH) and gamma-glutamyltransferase (GGT) enzymes. The diet given to these animals contained 36 mg/kg of Cu, with a Cu/Mb-ratio (CMR) of 16.6:1. In this case, it appears that younger animals were potentially more sensitive to increased Cu content within their diet. The Cu content within the livers of four other llamas from the same herd, whose death was reported to have other causes than poisoning, also reached levels up to 890 mg/kg DM, similar to those of the younger llamas that died from such a concentration according to those authors. It remains uncertain whether these animals might have also experienced undetected Cu poisoning, or if such elevated Cu concentrations are a common occurrence in llamas.

A case of subclinical accumulation of Cu in a 9-year-old male llama presented with depression and sternal recumbency was described by Weaver et al. in 1999 [26]. Again, necrosis histologically found in the liver also indicated Cu intoxication. The Cu content in the liver was reported to be 327 mg/kg wet weight (WW) for this animal. Liver biopsies of other animals in the herd showed Cu contents of 132–442 mg/kg WW even in clinically unremarkable animals. In addition to the increased Cu contents, an increased serum activity of GGT was found in all animals, whereas the serum activities of AST and serum sorbitol dehydrogenase (SDH) were not changed. Two different mineral feeds containing 46 and 280 mg/kg Cu were used in this stock.

In 2010, Fowler reported the case of a llama with a Cu plasma level of 0.83 μg/mL (= 13.03 μmol/L) showing clinical signs such as anorexia, icterus and haemoglobinuria in combination with haemolysis as atypical compared to the other reports in SACs mentioned above, but more similar to reports in sheep [27].

Cu, as a component of many different metallo-enzymes, is involved in various essential metabolic processes in mammals in general. According to Van Saun, the average daily Cu requirement for SACs is 0.15 mg/kg bodyweight (=BW) [28]. For SACs with a BW of 60 to 160 kg, the daily Cu intake should therefore be 9 to 24 mg. Considering the assumed DM intake of 1.25–1.5% of BW, this corresponds to 9–12 mg/kg DM of the animals’ diet [28]. Fowler suggests that the Cu content in the ration for camelids should be < 15 mg/kg [27]. Mo, known to reduce the absorption of Cu, should ideally maintain a CMR below 10:1 in the diet, as a ratio exceeding 16:1 can be associated with Cu poisoning [25]. In general, poisoning can be assumed from a content of over 35 mg/kg Cu in the ration of SACs [29]. There is only little information about the clinical signs of chronic poisoning in SACs in the literature so far. Unlike in sheep, poisoning in SACs does not necessarily result in the pronounced haemolytic crisis with haemolysis and icterus but is more likely to exhibit symptoms of liver insufficiency [23, 24, 27]. Indications for the current Cu concentration in the blood are provided by blood samples from serum and plasma, whereby the plasma concentration generally seems to be the better indicator, as Cu could get lost because of clots [27, 30, 31]. Several reference values for the Cu content in the plasma for SACs (see Table 1) are reported, ranging from 2.1 to 16.4 μmol/L (0.13 and 1.04 μg/mL) for alpacas and llamas depending on age, feeding and sex [32–34]. In general, different authors documented healthy SACs showing lower plasma Cu levels than sheep [35, 36]. Females tend to have higher plasma Cu levels than males, while animals over 1 year old typically have higher plasma Cu levels than younger animals [33]. In a study of 20 free-ranging guanacos (*Lama guanicoe*) from Argentina, Karesh et al. found a mean Cu concentration of 6 μmol/L in the plasma [37]. Examining the mineral status of an animal by obtaining liver biopsies provides more accurate information, also for Cu accumulation status in the liver and the current risk of developing poisoning symptoms [38]. The content in the liver is reported in SACs in different ways. Puls indicated a Cu concentration of 25–100 mg/kg (WW) as adequate and 250–400 mg/kg (WW) as toxic [29]. It can be assumed that Cu and Mo are stored in a similar concentration in the different liver lobes. In 2004, Anderson compared four different liver lobes of alpacas, the mean values for Cu concentration varying between 95 and 197 ppm and those for Mo varying between 1.2 and 1.4 ppm [38]. To the best of our knowledge, no literature provides information about Cu content in serum, plasma or liver tissue specifically for vicuñas (*Vicugna vicugna*). Consequently, the comparison with researched values for alpacas and llamas is the only option here.

**Table 1:** Reference values from various authors for the copper content in blood serum or plasma. Values by Pechová et al. represent the range taken from results, in which the plasma copper concentrations of 288 animals of different ages and both sexes were examined, minimum and maximum are shown, while the median of all animals was 7.53 μmol/L and the mean 7.64 μmol/L with a standard derivation of 1.90 [34].

			Alpaca		Llama	
Authors	Published in	Medium (μmol/L)	Male	Female	Male	Female
Foster et al. [32]	2009	Serum	2.1–12.5		6.1–7.9	
		Plasma	3.6–11.2		–	
Pechová et al. [33]	2018	Plasma	2.93–12.60	3.10–16.41	–	–
Stanitznig et al. [34]	2018	Serum	4.28–10.86	4.88–10.37	5.09–11.85	4.26–10.54

All values are given in micromoles per litre

## Case Presentation—Intoxication in a Vicuña

### Anamnesis

On 1 September 2018, a 3-year-old female vicuña (vicuña 1) from a zoological garden in northern Germany was presented to the clinic with suspected ileus. It had been housed in the enclosure since November 2016 in a group with three other vicuñas and two alpacas. The animal had refused feed intake for 1 day and was separated from the group to achieve better observability, having shown no defaecation during the previous 24 h. During the initial general examination on the following day on site, the animal was recumbent in sternal position and exhibited apathic behaviour, bruxism, sticky mucous membranes and atony of the gut compartments and intestines during auscultation. Further examination by palpation, auscultation and abdomainal ultra-sonography revealed unremarkable findings. Based on the diagnostic findings, initial therapeutic treatments for convulsive colic were initiated on site, consisting of the administering of a continuous intravenous drip infusion containing electrolytes and glucose of 1000 mL (Sterofundin®, VG-5) in addition to an antipyretic analgetic (intravenous 40 mg/kg body weight (BW), Metamizole®, WDT-Wirtschaftsgenossenschaft deutscher Tierärzte e.G., Garbsen, Germany), anti-inflammatory medication (intravenous 2 mL = 8 mg total dose Dexamethasone®, bela-pharm) and antibiotic treatment (intramuscular 5 mL = mg/kg BW: Pen–Strep, Penicillin-Dihydrostreptomycin 45 Mega ad us vet.®, Livisto). There was no improvement in the condition of the animal during several hours, which led to the decision to transfer the animal to the clinic for further diagnostic evaluation.

### Clinical Findings and Surgery

**Fig. 1:**
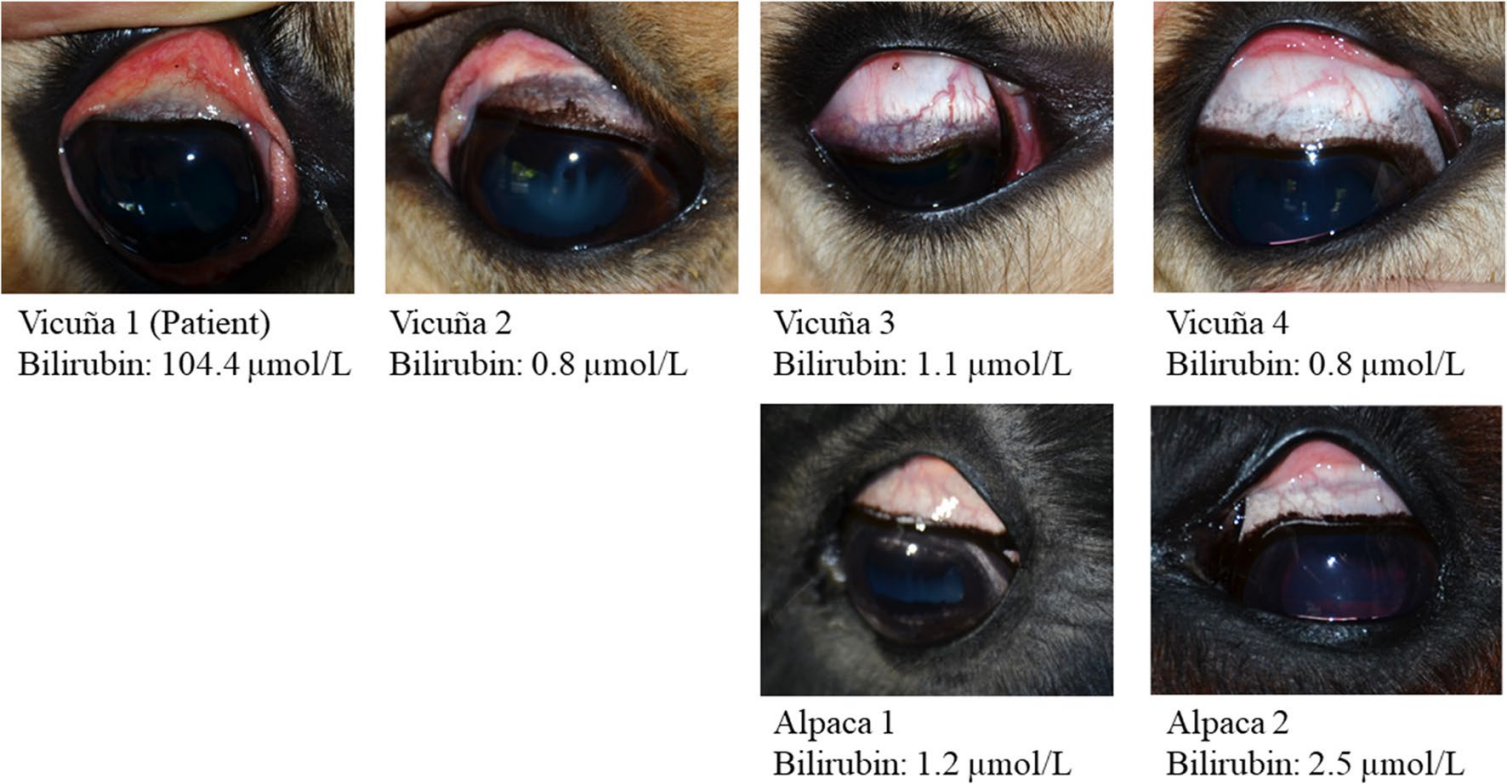
Photographs of sclera of the South American camelids in the zoo enclosure. Note the clear, icteric discolouration of the sclera as well as an increase in the serum bilirubin level of the patient compared to the other animals

On presentation to the clinic, the vicuña had a BW of 36 kg and exhibited a moderate body condition according to the classification of nutritional status at necropsy by Neubert et al. in 2022 [39]. The sclerae were icteric (Fig. 1) and the animal refused to stand due to weakness. Blood samples (EDTA, Monovette 9 mL K3E, Sarstedt AG & Co. KG, Nümbrecht, Germany; Lithium-Heparin, Monovette 9 ml LH, Sarstedt AG & Co KG; serum, S-Monovette 9 mL Z, Sarstedt AG & Co KG) were taken from the jugular vein for investigation of haematological and clinical chemistry parameters. Abdominal radiography, ultrasonography and faecal parasitological examination revealed no evidence of pathological changes. A diagnostic laparotomy was performed due to the unexplained colic symptoms. For this purpose, the animal was initially sedated with ketamine (Ketamidor® 100 mg/mL, WDT-Wirtschaftsgenossenschaft deutscher Tierärzte e.G., Garbsen, Germany) for intubation, and further inhalation anaesthesia was induced with isoflurane (Isofluran CP® 1 mL/mL, CP-Handelsgesellschaft GmbH, Burgdorf, Germany, dosage 1–3% in inhaled air). The animal was placed in a supine position and the abdominal cavity was opened in the *Linea alba*. Macroscopically, the abdominal cavity showed no pathological abnormalities. The rectum contained large, firm but deformable faeces, while most of the rest of the bowel was empty. Motility was reduced in all parts of the bowel. The third compartment (C3) of the gut was plump and filled, but the contents were deformable. The liver revealed sharply demarcated margins and a physiological consistency. The abdominal wall was closed with absorbable suture material, and an initial suspected diagnosis of ileus was excluded. After being transported back to the zoological garden under veterinary supervision, the animal regained clear consciousness and remained in ventral recumbency. The day after the vicuña was found dead.

### Investigation of the Herd

For checking the health status of the other animals in the herd, further blood samples (EDTA, Monovette 9 mL K3E, Sarstedt AG & Co. KG; Serum, S-Monovette 9 mL Z, Sarstedt AG & Co. KG) of the remaining vicuñas (vicuñas 2–4) and alpacas (alpaca 1 and 2) from the same enclosure were taken on 6 September. The mineral feed was checked for trace element contents (Table 2). Additionally, one of the remaining alpacas died 2 years later due to other reasons and the analysis of the Cu contents of the liver was also performed (Table 3). The mineral feed was checked for trace element contents (Table 2).

**Table 2:** Selection of relevant additives included in the mineral additive feedings for the SACs from the zoo

		Alpaka 17 Struktur®	VitaMiral®
Daily doses per animal		300 g	Fed quantities are not documented
Minerals	Unit	Quantity per kg	
E1 iron (Fe(II) sulphate, hydrated)	mg	28	–
E1 amino acids (Fe-chelate)	mg	30	–
E6 zinc (Zn-oxide)	mg	–	7000
E6 zinc (Zn(II)-sulphate, hydrated)	mg	115	–
E6 amino acids (Zn-chelate)	mg	28	–
E5 manganese (Mn-II-oxide)	mg	–	3300
E5 manganese (Mn(II)-sulphate, hydrated)	mg	113	–
E5 amino acids (Mn-chelate)	mg	25	–
E4 copper (Cu-II sulphate, pentahydrate)	mg	33	2500
E4 amino acids (Cu-chelate)	mg	9	–
E2 iodium (calcium iodate, hydrated)	mg	1.01	100
Cobalt (coated granulated cobalt (II) carbonate)	mg	0.9	25
E8 selenium (sodium selenite)	mg	0.3	50
Other additives	Unit	Quantity per kg	
Vitamin A	I.U.	14.000	1.000.000
Vitamin D3	I.U.	1400	125.000
Vitamin E	mg	250	5000
Vitamin B1	mg	6	–
Vitamin B2	mg	7.2	–
Vitamin B3	mg	9.00	–
Vitamin B6	mg	1.50	–
Vitamin B12	mcg	30	–

*I.U.* international unit, *mg* milligramme, *kg* kilogramme, *mcg* microgramme

**Table 3:** Haematological and clinical chemistry results of the vicuña patient (vicuña 1) and the remaining South American Camelids in the same enclosure measured in serum samples

		Reference	Vicuña 1 (patient)	Vicuña 2	Vicuña 3	Vicuña 4	Alpaca 1	Alpaca 2
Haematology								
WBC count	× 10^9^/L	8–16	12.8	7.9	11.7	14.5	8.1	9.1
WBC count (corrected)	× 10^9^/L		12.5	7.9	11.7	14.4	7.9	9.1
RBC count	× 10^12^/L	10.5–15	–	14.62	11.53	13.23	8.43	17.13
Haemoglobin	g/L	110–161	123	160	154	127	137	185
PCV	L/L	0.26–0.37	0.28	0.36	0.35	0.29	0.31	0.39
MCV	fL	22.8–26.3		25	30	24	37	23
MCH	pg	9.8–11.0		11	13	10	16	11
MCHC	g/L	411–454	439	444	440	438	442	474
Platelets	× 10^9^/L			327	373	378	271	195
Normoblasts	%		2	0	0	0.5	2	0
Lymphocytes	× 109/L	1.1–5.2	0.56	2.05	3.86	3.32	1.91	1.05
Segmented neutrophils	× 109/L	3.4–9.1	10.92	4.74	6.79	9.31	4.45	5.73
Banded neutrophils	× 109/L	0–0.1	0.75	0	0	0	0.16	0.46
Eosinophils	× 109/L	0.8–3.4	0.13	0.83	0.94	1.23	1.15	1.68
Basophils	× 109/L	0–0.2	0	0.04	0.06	0.22	0.04	0
Monocytes	× 109/L	0.2–0.9	0.19	0.24	0.06	0.36	0.24	0.18
Reticulocytes	1/1000			21	29	32	54	33
NLR		0.5–2.9^a^	**20.7**	2.3	1.8	2.8	2.4	5.9
Anisocytosis			+	+	+	+	+	+
Polychromasia			+	(+)	(+)	(+)	+	
Poikilocytosis			(+)					
Clinical chemistry								
Bilirubin total	μmol/L	0.2–1.01	**104.4**	1.38	1.1	0.81	2.48	1.21
Bilirubin direct	μmol/L		**41.87**					
Total protein	g/L	56.2–70.4	48.7	55.5	58.8	56	61.1	63.9
Albumin	g/L	28.4–37.4	35.5	49.7	46.1	34.3	42.3	43.6
Globulin/albumin			0.37	0.12	0.28	0.63	0.44	0.47
CK	U/L	43–276	**2023**	24	50	36	30	28
AST	U/L	30–144	**857**	82	84	75	71	56
CK/AST			2.4	0.3	0.6	0.5	0.4	0.5
GLDH	U/L	4–21.2	**150**	3	3	24	2	2
AP	U/L	30–144	8					
GGT	U/L	15–43	43	18	11	18	15	17
Creatinine	μmol/L	104–168	**667**	149	163	156	144	196
Urea	mmol/L	4.5–9.1	12.34	7.87	9.4	10.92	7.1	9.01
Glucose	mmol/L	5.4–7.3	28.97					
l-Lactate	mmol/L		40.1					
d-Lactate	mmol/L		0.31					
Calcium	mmol/L	2.1–2.5	2.51	2.49	2.41	1.76	2.54	2.7
Magnesium	mmol/L	0.8–1.1	1.63	0.78	0.82	0.63	0.82	1.04
Phosphate	mmol/L	1.1–2.8	1.18	1.72	1.86	1.72	1.96	0.87
Sodium	mmol/L	148–155	139.4	150.7	155.5	148	152.8	143.8
Potassium	mmol/L	4–5.2	3.13	3.99	4.6	5.39	5.66	4.75
Selenium	μg/L	15.7–206.3	42.9	184.3	174.8	181	200.1	179.6
Copper	μmol/L	Table 1	**33.4**	9.9	12.3	14.8	12.9	15.1
Ceruloplasmin	mg/L		**261**	95.2	105.5	146.6	106.9	153.4

Anomalies are in bold. *PCV* packed cell volume, *MCV* mean corpuscular volume, *MCH* mean corpuscular haemoglobin, *MCHC* mean corpuscular haemoglobin concentration, *NLR* neutrophil-to-lymphocyte ratio, *CK* creatine kinase, *AST* aspartate aminotransferase, *CK/AST* creatine-aminotransferase quotient, *GLDH* glutamate dehydrogenase, *AP* alkaline phosphatase, *GGT* gamma-glutamyltransferase. Reference values from Hengrave Burri et al. (2005) [40], except for NLR

^a^From Hajduk (1992) [41]

### Feeding

In addition to a daily ration of hay, branches and grass depending on availability, the SACs in the enclosure were given a supplement mixture of cereals and minerals with feed supplement containing Cu (Alpaka 17 Struktur®, Schroeder Futtermittel, Hofheim, Germany) in addition to low-dose cattle mineral feed twice a week (VitaMiral® Gruen, VitaVis GmbH, Minden, Germany). Moreover, different salt licks (producer not documented, containing sodium chloride (NaCl)) and mineral licks (producer not documented, “grey” containing NaCl, Cu, I (iodine), or “red” containing NaCl, I, Fe) were made accessible alternately or in parallel.

### Laboratory Findings

The examination of the blood samples was conducted in the clinic’s own laboratory, haematological analysis followed standard manual methods [42]. Examination of the parameters of clinical chemistry and trace element analysis was carried out as previously described using atomic absorption spectroscopy (AA Spectrometer M Series, Thermo Electron Ltd., Cambridge, UK) [43]. Due to a lack of reference values for vicuñas, reference values for female adult alpacas from Hengrave Burri et al. from 2005 were used for evaluating the results [40]. All blood results are listed in Table 3.

#### Vicuña 1 (Patient)

As the most prominent finding, serum Cu of the patient was significantly elevated compared to the reference limits provided in Table 1 and to other vicuñas and alpacas of the same enclosure. No reference values for ceruloplasmin for SACs were available. However, compared to the results of the other vicuñas and alpacas, the measured value appeared to be elevated. Clinical chemistry of the blood sample revealed pre- to intrahepatic icterus (hyperbilirubinaemia involving direct and indirect bilirubin), renal failure (azotaemia, hyponatraemia, hypokalaemia, hypomagnesaemia) and liver damage, as indicated by hypoproteinaemia, hyperbilirubinaemia of direct bilirubin and increased enzyme activities of CK (creatine kinase), AST and GLDH (glutamate dehydrogenase). Hyperglycaemia might be attributed to stress and pain. The activity for GGT approached the upper reference value. Serum selenium was within the reference values established by Stanitznig et al. [34] for female alpacas, but still lower than the selenium values of other SACs in the same enclosure. The main haematological findings of the patient were neutrophilia with band neutrophils and lymphopaenia, indicating acute inflammation. This was also reflected in a significantly increased neutrophil-to-lymphocyte ratio (NLR). Presence of normoblasts gave a hint of possible increased erythropoiesis.

#### Vicuñas 2, 3 and 4

Clinical chemistry showed high values for albumin for all vicuñas. In vicuña 3 and 4, plasma urea and plasma Cu concentrations were increased. Vicuña 4 exhibited increased activity of GLDH, hypocalcaemia, hypomagnesaemia and hyperkalaemia. Haematological investigation revealed no remarkable abnormalities for the remaining vicuñas except for low monocytes for vicuña 3 and the presence of normoblasts for vicuña 4.

#### Alpacas 1 and 2

Both alpacas had elevated albumin levels, hypercalcaemia and increased plasma Cu levels. Total bilirubin was slightly increased in alpaca 1. Haematological analysis of both alpacas revealed no remarkable abnormalities except for band neutrophils in both cases and the presence of normoblasts in alpaca 1. The NLR of alpaca 2 was moderately increased.

### Trace Elements in the Liver

The Cu content in the liver of vicuña 1 was elevated to 747.4 mg/kg (WW), while the content of Mo appeared slightly lower compared to reference values of other ruminants, since there were none found in literature for SACs (Table 3). In addition, elevated Cu levels were observed in liver analyses of an alpaca from the same enclosure, which was later euthanised in 2020, and a sheep from the same zoo, which was later slaughtered in 2020 (Table 4).

**Table 4:** Trace elements from the liver of the vicuña patient, compared with those from an alpaca and a sheep from the same zoo. Since no references for vicuñas were available, references from Puls [29] were given. The liver of vicuña 1 had a significantly increased copper content and significantly decreased molybdenum content. The copper levels of the other animals fed with the same mineral feed were also increased

		Zoo animals			Comparative animals (Puls 1994)			
Hepatic trace elements		Vicuña patient	Alpaca 2020	Sheep 2020	SACs	Sheep	Goats	Cattle
Copper	mg/kg (WW)	**747.4**	**235.3**	**123.7**	25–100	25–100	25–150	25–100
Selenium	mg/kg (WW)	0.435	–	–	n. a.	0.25–1.5	0.25–1.2	0.25–0.5
Cobalt	mg/kg (WW)	0.028	–	–	n. a.	0.020–0.085	n. a.	0.020–0.085
Molybdenum	mg/kg (WW)	**0.06**	–	1.88	n. a.	1.5–6.0	>0.31	0.14

*n. a.* not available, *SACs* South American camelids, *WW* wet weight

### Necropsy and Histological Findings

At necropsy, vicuña 1 was in moderate post-mortem preservation and a moderate nutritional state. Multifocal moderate oedaema was present in the subcutis. The pericardium contained 25 mL of a serosanguinous fluid, which was found to contain a high level of glucose (Combur^9^Test® for urinalysis). A total of 100 mL of a serosanguinous fluid was also detected in the abdomen. In addition, multifocal petechiae were located in the endocardium, pericardium, epicardium and pericardial fat tissue as well as perineurally on the brachial plexuses and sciatic nerves. The kidneys were light brown on both sides and of a pulpy consistency. A mildly elevated urea content of 120 mg/dL (reference < 50 mg/dL) was detected in the anterior eye chamber fluid. Other macroscopic findings included dried up ingesta components in the C3 compartment. Microbiological examination showed low levels of *Clostridium perfringens* in the small intestine. Using mass spectrometry analysis, no genes for major toxins including alpha toxin were detected.

**Fig. 2:**
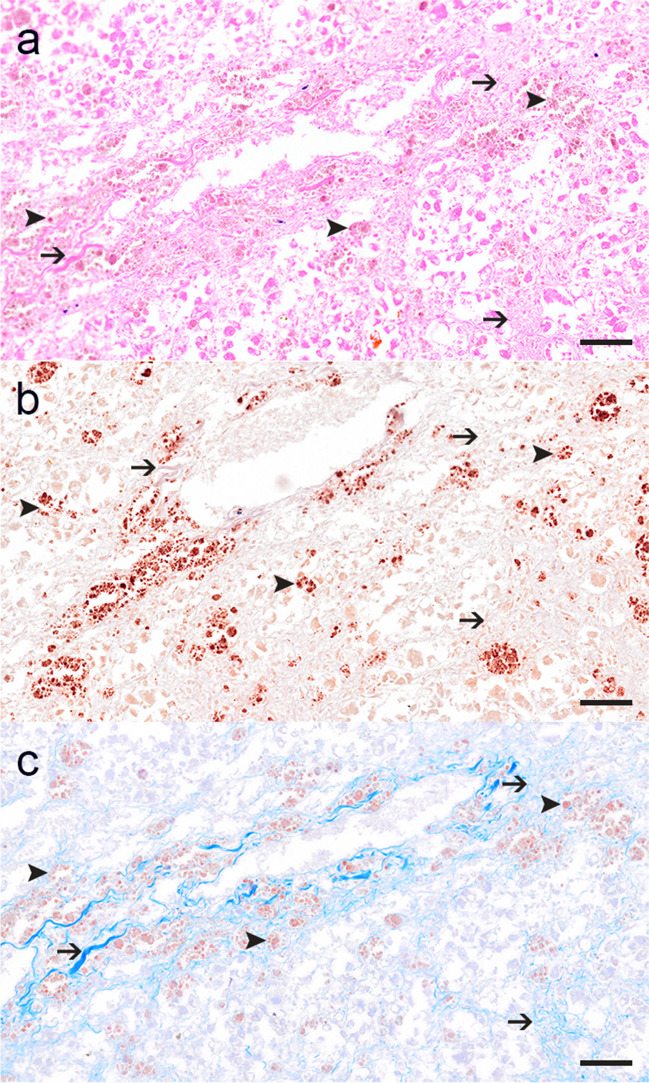
Illustration of the pathomorphological changes within liver tissue of vicuña 1 as assessed in light microscopic examination. Note the intracytoplasmic accumulation of coarse brownish pigment (arrowheads) of hepatocytes as well as an increased amount of fibrous tissue (arrows) already detectable in haematoxylin and eosin (HE; **a**) stained slides. The intracytoplasmic pigment (arrowheads) stains positive in rhodanine stain (**b**) and is often accompanied by variable amounts of collagen fibres (arrows) as best depicted in Heidenhain’s azan stain (**c**). Unfortunately, due to the poor state of preservation, necrosis could not be differentiated from autolysis. Bar = 50 μm

A representative organ spectrum was collected after necropsy and fixed in 10% neutral-buffered formalin. Tissue samples were subsequently embedded in paraffin wax, cut at approximately 3μm thickness and stained with haematoxylin and eosin (HE). Light microscopical examination revealed a periportally accentuated, multifocal, moderate, intracytoplasmic storage of a brown granular pigment in hepatocytes (Fig. 2a). The pigment stained red-brown on rhodanine stain (Fig. 2b) and was therefore identified as Cu. Additional histochemical stains for the detection of ferrous iron (Turnbull’s blue stain) and bile pigment (Fouchet stain) were negative. Histochemical visualisation of collagen fibres (Heidenhain’s azan trichrome stain) showed a mildly to moderately increased amount of connective tissue (Fig. 2c). Fibrosis was predominantly found within areas of Cu accumulation. The sternal bone marrow was cell-poor with occurrence of all three haematopoietic progenitor cell lines. Tonsils and mesenteric lymph node showed mild lymphoid hyperplasia. The oesophagus exhibited moderate, multifocal haemorrhages. Overall, histopathological examination was limited due to the moderate state of preservation. All other organs were unremarkable as far as assessable on histological examination.

## Discussion

In the context of our investigation, we dealt with a diagnosis of chronic Cu poisoning in a SAC. The literature has emphasised the importance of multifaceted approaches to ensure a diagnosis in patients of different species in general [1]. Vicuña 1 was initially presented with non-specific clinical signs, but the presence of icteric mucous membranes initiated further diagnostics. Highly increased Cu levels in serum (33.4 μmol/L) and liver samples (747.3 mg /kg WW) subsequently confirmed that the animal was suffering from chronic Cu intoxication, while further signs of haemolysis, as normally observed in sheep, were not present. The fact that there are only few reports of chronic Cu poisoning in SACs makes it difficult to confirm this diagnosis. The parallels observed between vicuña 1 and previously described reports include mainly physical condition with recumbency, apathy, colic symptoms and weakness, while the deterioration occurred within a short period of time in all cases. The Cu levels in serum and liver samples of the investigated patient were within the range of those observed in other cases where animals died. Additionally, when considering the concentration in WW liver tissue compared to DW methods as in the other cases, it becomes apparent that the Cu levels would be even approximately four to five times higher.

Regarding other findings, the patient’s serum values of bilirubin were 104.4 μmol/L for total bilirubin and 41.87 μmol/L for conjugated bilirubin, which results in a value of 62.53 μmol/L for unconjugated bilirubin. Comparative data from healthy alpacas, as reported by Flores et al. in 2016, indicate severe increases in both conjugated and unconjugated bilirubin in the patient: total bilirubin with 0.62 ± 0.51 mg/dL (= 10.54 ± 8.67 μmol/L); direct bilirubin with 0.13 ± 0.09 mg/dL (= 2.21 ± 1.53 μmol/L); indirect bilirubin with 0.51 ± 0.52 mg/dL (= 8.67 ± 8.84 μmol/L) [44]. While there is no other evidence of haemolysis, the significantly increased serum bilirubin level (104.4 μmol/L), with less than half being direct bilirubin (41.87 μmol/L), indicates pre- to intrahepatic icterus. The latter aligns with a haemolytic crisis commonly observed in sheep suffering from chronic Cu intoxication, but contrasting with the findings of previously mentioned reports in SACs, and supporting Fowler’s assertion that clinical signs in SACs differ from classical symptoms in sheep [27]. One should also consider possible fasting hyperbilirubinaemia as previously described in cattle [45] and horses [46]. However, this possibility is of limited relevance in this case as the observed bilirubin levels, also conjugated bilirubin, were well above the established ranges and there was no association with hypoalbuminaemia. At the same time, assessment of necrotic liver changes was made difficult by marked autolysis of the organ. Consequently, the aetiology of the hyperbilirubinaemia in this case is still insufficiently clarified.

Regarding ceruloplasmin, the serum concentration was significantly higher compared to the other SACs in the enclosure. This aligns with the findings of Blakley and Hamilton who found strong correlations between ceruloplasmin oxidase activity and elevated serum or plasma Cu concentrations in cattle and sheep [47]. Ceruloplasmin also plays a role as an acute-phase protein and has been shown to be elevated in ruminants after stressful situations such as animal transport [48], which should not be disregarded here.

Another haematological deviation in the affected vicuña was also a significantly increased NLR, which did not deviate from the reference value in the other animals in the enclosure, with the exception of alpaca 2. Little is known about NLR in SACs to date. In other species, NLR is used as a prognostic marker for various diseases [49, 50] or as an indicator of stress [51, 52]. There is also evidence that NLR may be associated with stress in SACs [53]. It remains unclear whether the increase in NLR in the present case was due to inflammation or stress, although it is likely that both factors contributed. The increase in NLR in alpaca 2 remains unclear.

For the values presented here, it should be noted that the literature recommends the taking of plasma samples as opposed to serum samples for determining Cu levels. Besides, liver Cu values from biopsies or post-mortem samples are considered more reliable for diagnosis [27]. Liver biopsies from living animals can be conducted without significant clinical effects [54] to exclude suspicion of Cu poisoning. However, it is unusual to find markedly high Cu levels in only one animal if the intoxication is solely attributed to high Cu content in the feed ration. Either vicuña 1 displayed an individually heightened propensity for Cu accumulation or the animal was exposed to additional sources of Cu intake. Interestingly, the Cu levels in serum and liver samples of other animals from the same enclosure, although not as high as in our patient, were also slightly high. In addition, the combination of over-dosed Cu levels in the ration and the suffering appearing in only one animal in a herd is not the first observed case, as a similar case was also previously reported by Carmalt et al. in 2001 [24]. Also, the literature suggests that Cu intoxications may be caused, among other things, when there is a deficiency in antagonistic elements such as Zn, Mb, Fe or Ca, as Cu absorption tends to increase in the absence of these [1]. Although reference values for Mb content in the liver of SACs are lacking, a noticeable discrepancy is observed when comparing the concentrations in Table 3, as they are strikingly low compared to other ruminant species. Mb is described in connection with low or raised Cu levels in the literature, while the authors also describe a CMR in the feed of ruminants, which should be observed to prevent Cu poisoning [55, 56]. In our case, as Mb was not explicitly listed in the mineral concentrate of the feed, this omission could also have led to an imbalance in the feeding ration, consequently increasing Cu absorption. Moreover, it is not entirely clear how feeding ration data from other camelids can be transferred to vicuñas. The necropsy results provided no further insights into the cause of death of the animal. According to Fowler, liver necrosis and nephrosis are the most important post-mortem lesions associated with Cu intoxication [27]. Based on rhodanine staining, the liver of the animal showed a moderate degree of Cu storage, in combination with an increased amount of fibrous connective tissue, which could be indicative of chronic liver damage. Moreover, the distribution pattern of Cu storage also hints at a more chronic process. Unfortunately, due to the moderate state of preservation, corresponding necrosis could not be verified.

The role of the low detection of *Clostridium perfringens* in the small intestine during necropsy is difficult to classify here, while the detection is additionally supported by increased glucose content in the pericardial effusion as commonly observed in association with the kidney disease of sheep caused by infections with *Cl. perfringens* type D [57, 58]. This disease has only been described sporadically in camelids without the measurements of glucose in pericardial effusion so far [59]. The presence of petechia in various layers of the myocardial wall, peripheral nerves and the oesophagus could indicate enterotoxaemia caused by pathogens such as clostridia, although similar changes can also occur in other toxic or primarily vascular changes [60, 61]. Additionally, these changes may have also occurred agonal [61]. Also, the strain carried no genes for major-toxins including alpha-toxins and *Cl. perfringens* is regularly detected in ingesta of the small intestine of healthy animals [62, 63].

The renal changes, as indicated by azotaemia, slightly elevated urea levels in the anterior eye chamber, and the autolytic consistency of the kidneys in the necropsy are also not conclusively clarified here, as urea in the eye chamber can also have pre-renal causes [64, 65]. In general, during a haemolytic crisis caused by Cu poisoning, there can be an accumulation of excess haemoglobin in the renal tubes, which can potentially lead to tissue damage [22]. Additionally, the presence of a potential clostridiosis, as previously mentioned, might contribute to the autolysis of the kidneys [57]. Both of the discussed scenarios could be linked to possible kidney changes. Due to the poor ability to exam the kidneys post mortem, this point remains unclear.

## Conclusion

In summary, the precise cause of death in the vicuña remains elusive due to the overall non-specific nature of the exhibited symptoms. Nevertheless, the animal exhibited significantly elevated Cu levels in both serum and liver, which align with the manifestations of Cu poisoning as described in the literature. These signs include an increase in both conjugated and unconjugated bilirubin, resulting in icterus, along with symptoms observed in chronic Cu poisoning in other ruminants: recumbency, reduced appetite, lethargy, colic, elevated liver enzyme activity and potential renal failure. While certain identified signs may also manifest in sheep affected by clostridiosis, the authors therefore are inclined to interpret the cause of death as either Cu poisoning, or a combination of factors including Cu poisoning as being the most likely cause of death.

## Data Availability

The datasets analysed during the current study are not publicly available due to data protection regulations, but information are available from the corresponding author on reasonable request.

## Abbreviations

AP: Alkaline phosphatase
AST: Aspartate aminotransferase
BHV-1: Bovine herpesvirus-1
BTV: Bluetongue virus
BVDV: Bovine viral diarrhoea virus
BW: Body weight
CK: Creatinine kinase
CK/AST: Creatine-aminotransferase quotient
CMR: Copper-to-molybdenum ratio
Cu: Copper
DM: Dry matter
EDTA: Ethylenediaminetetraacetic acid
Fe: Iron
GGT: Gamma-glutamyltransferase
GLDH: glutamate dehydrogenase
HE: Haematoxylin and eosin
I: Iodine
LDH: Lactate dehydrogenase
MCH: Mean corpuscular haemoglobin
MCHC: Mean corpuscular haemoglobin concentration
MCV: Mean corpuscular volume
Mg: Magnesium
Mn: Manganese
Mo: Molybdenum
NaCl: Sodium chloride
NLR: neutrophil-to-lymphocyte ratio
NSAID: Non-steroidal anti-inflammatory drug
PCR: Polymerase chain reaction
PCV: Packed cell volume
RNA: Ribonucleic acid
S: Sulphur
SAC(s): South American camelid(s)
WW: Wet weight
Zn: Zinc
